# Multiplex PCR Identification of *Aspergillus cristatus* and *Aspergillus chevalieri* in Liupao Tea Based on Orphan Genes

**DOI:** 10.3390/foods11152217

**Published:** 2022-07-26

**Authors:** Zhong Wang, Qifang Jin, Qin Li, Xingchang Ou, Shi Li, Zhonghua Liu, Jian’an Huang

**Affiliations:** 1 Key Laboratory of Tea Science of Ministry of Education, Hunan Agricultural University, Changsha 410128, China; zwang@stu.hunau.edu.cn (Z.W.); qifangjin10@126.com (Q.J.); liqinvip@126.com (Q.L.); xco118@sina.com (X.O.); lishidodo@163.com (S.L.); zhonghua-liu@hunau.edu.cn (Z.L.); 2 National Research Center of Engineering and Technology for Utilization of Botanical Functional Ingredients, Hunan Agricultural University, Changsha 410128, China; 3 Co-Innovation Center of Education Ministry for Utilization of Botanical Functional Ingredients, Hunan Agricultural University, Changsha 410128, China; 4 Key Laboratory for Evaluation and Utilization of Gene Resources of Horticultural Crops, Ministry of Agriculture and Rural Affairs of China, Hunan Agricultural University, Changsha 410128, China

**Keywords:** *Aspergillus*, Liupao tea, orphan genes, multiplex PCR, rapid identification

## Abstract

“Golden flower” fungi in dark tea are beneficial to human health. The rapid identification method of “golden flower” fungi can verify the quality of dark tea products and ensure food safety. In this study, 6 strains were isolated from Liupao tea. They were respectively identified as *A. cristatus*, *A. chevalieri*, and *A. pseudoglaucus*. *A. pseudoglaucus* was reported as Liupao tea “golden flower” fungus for the first time. It was found that the ITS and BenA sequences of *A. cristatus* and *A. chevalieri* were highly conserved. It is difficult to clearly distinguish these closely related species by ITS sequencing. To rapidly identify species, multiplex PCR species-specific primers were designed based on orphan genes screened by comparative genomics analysis. Multiplex PCR results showed that orphan genes were specific and effective for the identification of *A. cristatus* and *A. chevalieri* isolated from Liupao tea and Fu brick tea. We confirmed that orphan genes can be used for identification of closely related *Aspergillus* species. Validation showed that the method is convenient, rapid, robust, sequencing-free, and economical. This promising method will be greatly beneficial to the dark tea processing industry and consumers.

## 1. Introduction

“Golden flower” fungi are important microorganisms in the fermentation process of dark tea, which can profoundly affect the quality, functional components and health efficacy of dark tea [1,2,3,4]. The mainly dominated “golden flower” fungal community in Fu brick tea was identified as *A. cristatus* [1,5]. *A. cristatus*, a promising probiotic candidate, is able to regulate the imbalance of intestinal flora [6,7]. In the past, the research on “golden flower” fungi mainly focused on Fu brick tea. Some Liupao tea would also form “golden flower” characteristics during the aging process. Previous studies have shown that there may be a variety of closely related species in the dark tea “golden flower” fungi [8]. In the post-fermentation processing of dark tea, the fermentation conditions of various “golden flower” fungi are different. Some fungi are potential mycotoxin producers in the microbiota of the post-fermentation and aging process [9,10,11]. Fungal contamination may produce toxins, such as aflatoxins, ochratoxin A, fumonisins, zearalenone and citrinin [12,13]. Mycotoxins pose a threat to consumer health. Comparative genomic analyses of *A. cristatus* indicated the absence of common mycotoxins biosynthetic gene clusters [14]. It is necessary to identify the species of “golden flower” fungi in Liupao tea.

At present, a variety of microbial species identification methods have been developed by using biomarkers based on biological macromolecules and metabolites, such as protein, DNA, lipids, volatile small molecules and other biomarkers [15,16]. Identification methods based on proteins or metabolites often need to rely on expensive large-scale precision instruments. DNA molecular marker methods are the most widely used methods for species identification. A variety of DNA application methods have been developed, such as loop-mediated isothermal amplification (LAMP), rolling circle amplification (RCA), PCR, RFLP and sequencing technology [15,17,18]. As the price of NGS sequencing has fallen, the technology has become the gold standard for microbial research [19,20]. However, there are still many barriers to practical application. First, many laboratories are still limited by bioinformatics and computational resources to analyze and process high-throughput data generated by NGS methods. Secondly, there are still many restrictive conditions for the application of NGS method in rapid detection scenarios. In the large-scale rapid detection of microorganisms involving public health and food safety, PCR identification method is still an important direction for the development of rapid detection methods. In recent years, multiplex PCR, real-time PCR, genome-based specific sequence amplification and other methods have been developed for rapid microbial species identification [21,22,23]. Fungal species are generally identified by multi-locus sequence analysis, usually using the housekeeping gene ITS, BenA, CaF and RPB2 [24,25]. However, the housekeeping gene sequences are highly conserved in some closely related species, and it is difficult to distinguish species clearly, even relying on sequencing technology. Therefore, it is necessary to develop new methods for identification of closely related species.

Comparative genomics analysis has been demonstrated to be able to distinguish *Lactobacillus* strains based on gene family analysis [22,26]. Orphan genes are species-specific sequences that have no homology to sequences in other evolutionary lineages [27,28]. According to the characteristics of orphan genes, they can be screened by comparative genomics analysis and used for rapid species identification. Now, we can use bioinformatics to find orphan genes through the large amount of genomic data available in public databases.

## 2. Materials and Methods

### 2.1. Sample Collection and Preparation

One hundred representative samples of Liupao tea circulating in the market were collected. All samples were evaluated by six well-trained panelists according to the Methodology of Sensory Evaluation of Tea (GB/T 23776-2009). Liupao tea samples with remarkable “golden flower” characteristics were selected; that is, yellow fungi or spores were distributed on the samples. Fu brick tea from different districts were collected.

### 2.2. “Golden Flower” Fungi Isolation

The isolation procedure of “golden flower” fungi was modified according to the previously reported method [5,29]. Briefly, each sample (5.0 g) was transferred to an Erlenmeyer flask containing sterile water (95.0 mL) and sterile glass beads, vortexed at 200 rpm for 30 min at 25 °C. A dilution plating technique was applied to isolate and purify “golden flower” fungi. Typical single colonies were purified using the streak plate method twice in succession. The obtained single colonies were regarded as purified colonies and inoculated into PDA slant test tubes. After culturing at 28 °C for 5 days, tubes were transferred to 4 °C for storage. 

### 2.3. PCR Amplification and DNA Sequencing

The direct colony PCR method was modified and used to amplify genomic sequences [30]. A pin point of purified fungal tissue was picked up and added to 60 μL of sterile water to form a suspension. Suspension was incubated at 95 °C for 10 min to release the DNA and centrifuged at 4000 rpm for 1 min, and the supernatant was used directly as the DNA template for PCR amplification. The volume of 25 μL of PCR reaction mixture consisted of 12.5 μL of 2 × Phanta^®^ Max Master Mix (Vazyme Biotech Co., Ltd., Nanjing, China), 3 μL of DNA template, 1 μL of 10 pmol/μL respective primers, and 7.5 μL of sterile water. The rDNA-ITS-region (ITS) sequences of the strains were amplified with universal primers ITS1 and ITS4, and the beta tubulin (BenA) DNA sequences were amplified with primers Bt2a and Bt2b, described by Liu et al. [31]. PCR conditions were optimized as follows: initial denaturation for 5 min at 95 °C, followed by 38 cycles of denaturation for 15 s at 95 °C, annealing for 20 s at 55 °C, extension for 1 min at 72 °C, and a final extension step for 5 min at 72 °C (modified from Peterson [24]). Positive controls were genomic DNA extracted from *A. cristatus*, *A. chevalieri* and *A. pseudoglaucus*, and a no template control was used as a negative control.

Genomic DNA was extracted using the Plant Genomic DNA Kit (Cat#DP305, Tiangen Biotech Co., Ltd., Beijing, China). The amplicons were sequenced by Sangon Biotech Co., Ltd. (Shanghai, China). Then, the obtained sequences were compared with available sequences in the NCBI database using the BLASTN programs and deposited in the NCBI GenBank database.

### 2.4. Phylogenetic Analysis

The above obtained sequences were aligned using the ClustalX program [32]. The Partition Homogeneity test was applied to the combined sequence of ITS and BenA from each species [33]. Non-informative characters of combined sequence were excluded [34]. The *p*-values threshold for significant incongruence was 0.01 in the Partition Homogeneity test [35]. Phylogenetic analysis of the sequences was carried out using Neighbor-Joining method performed with the MEGA [36]. 

### 2.5. Orphan Genes Screening

The genome and predicted protein sequences of *A. cristatus* (PRJNA271918), *A. chevalieri* (PRJDB10979), *A. glaucus* (PRJNA169684), *A. niger* (PRJNA19275), *A. fumigatus* (PRJNA131), *A. nidulans* (PRJNA130) and *A. flavus* (PRJNA606291) were downloaded from the NCBI website. The orphan gene identification method refers to the method described by Jiang et al. [37]. To identify orphan genes, the predicted protein sequences of *A. cristatus* or *A. chevalieri* were used as a query file to align in the predicted proteomes (excluding itself) of *A. cristatus*, *A. chevalieri*, *A. glaucus*, *A. niger*, *A. fumigatus*, *A. nidulans* and *A. flavus* by BLASTP, then used to search against the genome (excluding itself) of *A. cristatus*, *A. chevalieri*, *A. glaucus*, *A. niger*, *A. fumigatus*, *A. nidulans* and *A. flavus* by TBLASTN for gene sequences that might not be annotated. All protein sequences of *A. cristatus* or *A. chevalieri* with no homologs in previous searches (*E* value cutoff of 1 × 10^−5^) were extracted and further used as queries to search against the NCBI database (excluding genome of the query species) by BLASTP. The proteins detected no homologous sequences in previous searches (*E* value cutoff of 1 × 10^−5^) were considered as orphan proteins of the species. These coding sequences of orphan proteins were described as orphan genes in the species.

### 2.6. Specific Primers Designing and Multiplex PCR Amplification

Orphan gene sequences of *A. cristatus* and *A. chevalieri* were extracted from the genome to develop the specific primers. Primer3 was adopted to carry out primers based on the obtained orphan gene according to the primer design principles. To detect mismatch in genomes of closely-related species, selected primers were checked by the Primer Check (Simple e-PCR) program [38]. The specificity of the designed primer pairs was tested by PCR using DNA of *A. cristatus* and *A. chevalieri* strains isolated from different tea samples. Genomic DNA extracted from target species was used as the positive control, DNA extracted from non-target species was used as the negative control, and sterile water was used as the no template control for individual PCR reactions.

Multiplex PCR was developed for identification of *A. cristatus* and *A. chevalieri* using the species-specific primer sets based on orphan genes. The primer sets were mixtures of assigned primers. The volume of 35 μL of multiplex PCR reaction mixture consisted of 17.5 μL of 2 × Taq Plus Master Mix Ⅱ (Vazyme Biotech Co., Ltd., Nanjing, China), 3 μL of each DNA template, 1.25 μL of 10 pmol/μL respective primers, and up to 35 μL of sterile distilled water. PCR conditions were optimized as follows: initial denaturation for 5 min at 95 °C, followed by 38 cycles of denaturation for 15 s at 95 °C, annealing for 20 s at 63 °C, extension for 35 s at 72 °C, and a final extension step for 5 min at 72 °C. The PCR products were subjected to analysis by 2% agarose gel electrophoresis and visualized in a gel documentation system.

## 3. Results

### 3.1. PCR Success from Fungi Colonies

**Figure 1:**
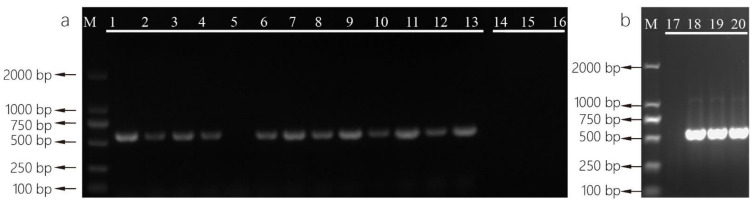
Amplification of rDNA-ITS-region of *Aspergillus* spp. colonies. Lane M: 2000 bp ladder, lanes 1–13: colonies incubated at 95 °C for 10 min, lanes 14–16: colonies unincubated, lane 17: negative control, lanes 18–20: DNA extracted from *A. cristatus*, *A. chevalieri*, and *A. pseudoglaucus* were used as positive controls.

Fungi strains isolated from Fu brick tea and Liupao tea samples were used as direct colony PCR templates. PCR amplification success rate was high when colonies incubated at 95 °C for 10 min were used as templates. On the other hand, colonies could not be successfully amplified without incubation (Figure 1). Subsequently sequencing results showed that the successfully amplified strains were identified as *A. cristatus*, *A. chevalieri*, and *A. pseudoglaucus*. To our knowledge, this is the first report that rapid direct colony PCR has been successfully applied to *Aspergillus* fungi. 

### 3.2. Identification of “Golden Flower” Fungi by Multilocus Sequence

The dominant strains of “golden flower” fungi isolated from tea samples were identified as *A. cristatus*, *A. chevalieri* and *A. pseudoglaucus* (Table 1), according to the results of ITS and BenA sequences analysis. The ITS sequences of the identified *Aspergillus* species showed high similarity (above 98%), while the similarity of *A. cristatus* and *A. chevalieri* was higher than 99%. Due to the high similarity of ITS sequences, the isolates could only be identified as *Aspergillus* species. Next, each strain can be identified to species level based on the BenA gene sequences similarity above 99%. The isolated strains were identified as *A. cristatus*, *A. chevalieri* and *A. pseudoglaucus*, respectively.

*A. cristatus*, *A. chevalieri* and *A. pseudoglaucus* were identified from “golden flower” Liupao tea, however only *A. cristatus* and *A. chevalieri* were isolated from Fu brick tea. *A. cristatus* was the dominant fungi in Fu brick tea collected from different districts in China. 

### 3.3. Phylogenetic Analysis

**Figure 2:**
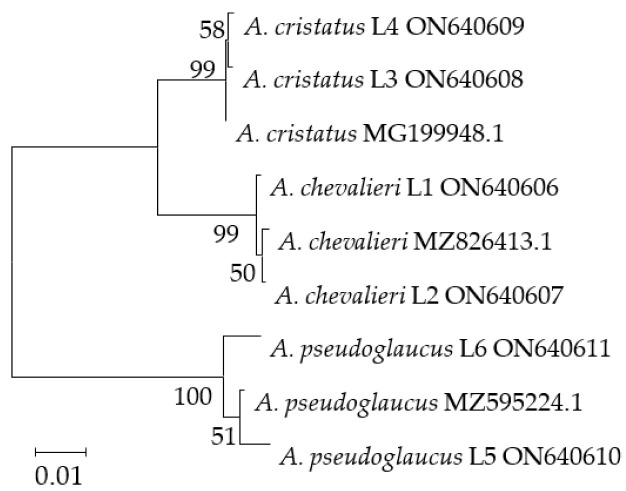
Neighbor-Joining phylogeny tree derived from *Aspergillus* spp. BenA sequences.

BenA sequences were selected for phylogenetic analysis according to the result of Partition Homogeneity test of BenA and ITS genes (*p* = 0.01). The phylogenetic tree derived from the BenA sequences exhibits three different clusters that are composed of strains belonging to three *Aspergillus* species (Figure 2). The phylogenetic tree showed that the relationship between *A. cristatus* and *A. chevalieri* was closer. 

### 3.4. Designing Primers Based on Orphan Genes

Through BLASTP and comparative genomics analysis, 42 and 59 orphan genes were respectively selected from the genome of *A. cristatus* and *A. chevalieri*. Some sequences were rather short, and those were omitted and not used for designing primer. The obtained primers (Table 2) were preliminarily tested by e-PCR using genome of *A. cristatus*, *A. chevalieri*, *A. glaucus*, *A. niger*, *A. fumigatus*, *A. nidulans* and *A. flavus*. The results of e-PCR showed that some of the primers occurred non-specific amplification (Table 3). However, the length of the amplification products was much longer than the length of the expected products. Therefore, non-specific amplification can be limited by limiting the extension time in PCR amplification.

**Figure 3:**
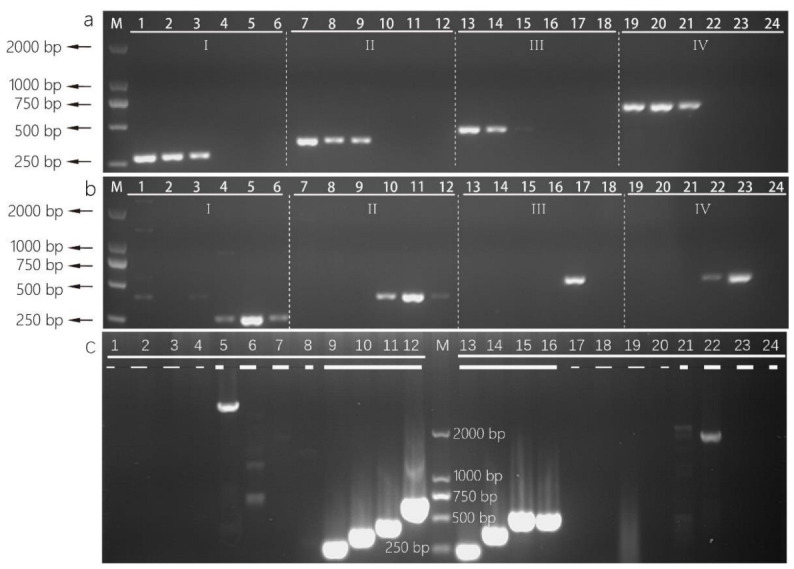
Evaluation of specificity of primers based on orphan genes from *Aspergillus* spp. (**a**) Primers used in lane groups I-IV, respectively, are aOP1, aOP2, aOP3 and aOP4; (**b**) primers used in lanes groups I-IV respectively are iOP1, iOP2, iOP3, iOP4. Lane M: 2000 bp ladder, samples in lanes group, I-IV respectively, are *A. chevalieri* L1, L2 and H7 and *A. cristatus* L3, L4 and H6; (**c**) negative controls and positive controls for each pair of primers; thin dotted line, respectively, represents no template controls, bold dotted line represents negative controls, bold line represents positive controls, lanes 1–12 respectively, are primers aOP1, aOP2, aOP3 and aOP4 and for target *A. chevalieri*, lanes 13–24 respectively, are primers iOP1, iOP2, iOP3 and iOP4 for target *A. cristatus*.

Four pairs of species-specific primers (Table 2) were selected for PCR verification. The expected length bands were amplified with aOP1, aOP2, aOP3 and aOP4 from the genomic DNA of various *A. chevalieri* strains isolated from different tea samples, and no band occurred when the genome of *A. cristatus* was used as DNA template (Figure 3a). However, the PCR results of iOP1 primers showed a mismatch band when *A. chevalieri* DNA or *A. cristatus* DNA was used as the template (Figure 3b). Those interference bands whose length were different from the expected bands can be clearly distinguished from the expected bands on the electropherogram. The PCR results showed that the amplification efficiency of primers iOP3 and iOP4 was different among different strains. In summary, primers based on orphan genes were effective and specific in distinguishing *A. chevalieri* and *A. cristatus*.

### 3.5. Multiplex PCR Amplification

**Figure 4:**
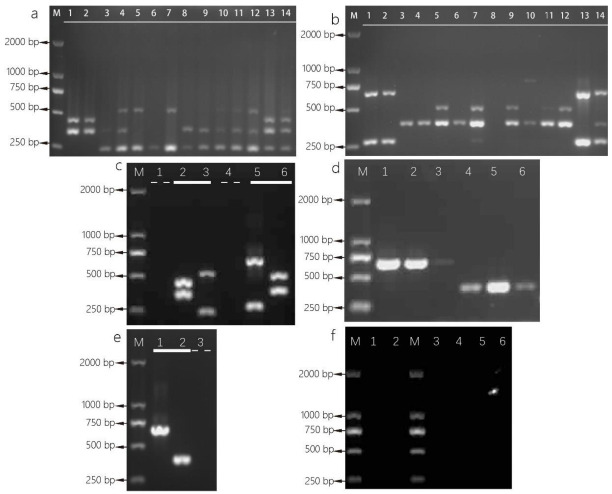
Multiplex PCR of *Aspergillus* strains. (**a**) Primer set 1 mixed aOP2, aOP3, iOP1 and iOP3; (**b**) primer set 2 mixed aOP1, aOP4, iOP2, and iOP4; lane M: 2000 bp ladder, lanes 1–2: *A. chevalieri* L1, L2, lanes 3–7: *A. cristatus* H1–H5, lane 8: *A. cristatus* H8, lane 9: *A. cristatus* S1, lane 10: *A. cristatus* Z1, lanes 11–12: *A. cristatus* L3–L4, lanes 13–14: artificial microbial communities M1–M2; (**c**) negative controls and positive controls for multiplex PCR, thin dotted line respectively are no template controls, bold line respectively are positive controls, lane 1: negative control for primer set 1, lane 2: *A. chevalieri* DNA was used as positive control for primer set 1, lane 3: *A. cristatus* DNA was used as positive control for primer set 1, lane 4: negative control for primer set 2, lane 5: *A. chevalieri* DNA was used as positive control for primers set 2, lane 6: *A. cristatus* DNA was used as positive control for primer set 2. (**d**) Primers set S containing aOP4 and iOP2 for *Aspergillus* identification; lane M: 2000 bp ladder, lanes 1–3: *A. chevalieri* L1, L2, H7, lanes 4–6: *A. cristatus* L3, L4, H6; (**e**) negative control and positive controls for aOP4 and iOP2 mixture amplification, lane 1: using *A. chevalieri* DNA as positive control, lane 2: using *A. cristatus* DNA as positive control, lane 3: negative control. (**f**) tea samples direct amplification, lanes M: 2000 bp ladder, lanes 1–2: using primer set S, lanes 3–4: using primer set 1, lanes 5–6: using primer set 2, lanes 1, 3, 5: tea sample containing *A. chevalieri* L1, lanes 2, 4, 6: tea sample containing *A. cristatus* L3.

Eight pairs of primers from *A. chevalieri* and *A. cristatus* were assigned to two primer sets, and the primers from each set were mixed for multiplex PCR (Table 2). Direct colony multiplex PCR were successfully amplified by using primers sets (Figure 4). The results of multiplex PCR showed that two bands of expected length could be amplified from various *A. chevalieri* strain DNA templates (Figure 4). However, the amplification results of *A. cristatus* DNA from different strains were diverse. The shorter expected amplification products of *A. cristatus* appeared, and the longer expected amplification products were only partially observed. An interference band whose length was similar to the expected band of *A. chevalieri* was amplified with primer set 1 from the genome of *A. cristatus* (Figure 4a).

Primers derived from *A. chevalieri* still amplified two expected bands in multiplex PCR when using artificial microbial communities M1 and M2 DNA as template (Figure 4). Only shorter bands of approximately 242 bp or 383 bp indicated *A. cristatus* were observed in artificial microbial communities multiplex PCR results, while expected bands indicative of *A. cristatus* were not observed in artificial microbial communities M1 PCR using primers set 2 (Figure 4). The electrophoretic bands of different species can be used for species identification.

Furthermore, we selected a pair of primers from each species for identification. The primers aOP4 and iOP2 were chosen and mixed to identify *A. chevalieri* and *A. cristatus*. Mixture of the primers amplified expected bands for *A. chevalieri* and *A. cristatus* colony PCR (Figure 4d). These bands of approximately 659 bp indicated *A. chevalieri*, and bands of approximately 383 bp indicated *A. cristatus.* However, false negative results were obtained when aOP4 and iOP2 were mixed to amplify tea samples containing target microorganisms (Figure 4f). This also occurred when primer set 1 and primer set 2 were used for tea sample amplification (Figure 4f). The low abundance of DNA in tea samples may cause false negative results. Tea polyphenols and other secondary metabolites in tea samples may also inhibit PCR amplification.

## 4. Discussion

### 4.1. Advantages of Direct Colony PCR for Aspergillus

Our direct colony PCR worked well in 16 *Aspergillus* strains isolated from tea samples. This method is also suitable for subsequent multiplex PCR. Template preparation for direct colony PCR amplification requires no DNA extraction and only cost 10 min for rapid fungal lysis. However, the previously reported direct PCR of *Aspergillus* using a commercial kit required 1 h of fungal lysis [39]. In this method, high-throughput preparation of DNA templates can be achieved by using 96-well plate incubation. This will greatly improve the efficiency in large-scale detection, and this PCR method does not require pre-treatment of fungi colonies described by [40]. In our experiment, *Aspergillus* colonies directly added into the PCR reaction mixture failed to amplify despite previous success in *Cladosporium*, *Geomyces*, *Fusarium* and *Mortierella* [30]. This direct colony PCR method is suitable to identify *Aspergillus* strains and obtain sequences from *Aspergillus*.

### 4.2. “Golden Flower” Fungi in Liupao Tea

In our study, the dominant strains of “golden flower” fungi isolated from Liupao tea samples were identified as *A. cristatus*, *A. chevalieri* and *A. pseudoglaucus*. This is the first time that *A. pseudoglaucus* has been isolated from Liupao tea. Some yellow/orange pigmented fungi growing on the ham surface were identified as *A. pseudoglaucus* [41]. Correlation analysis revealed that *A. pseudoglaucus* showed significant positive correlations with key volatile compounds of traditional dry sausages [42,43]. The enzymatic properties of *A. pseudoglaucus* were different from those of *A. cristatus* and *A. chevalieri* [44]. *A. pseudoglaucus* may also play an important role in the formation of Liupao tea quality. Mao et al. (2017) found that *Eurotium* was widespread in Liupao tea, and identified as *E. amstelodami*, *E. niveoglaucum*, *E. repens*, *E. rubrum*, *E. tonophilum* and *E. cristatum* [45]. The results of different studies were quite different, indicating that the aging environment of Liupao tea may affect the fungal community. It was found that the amount of *Eurotium* sp. plays a vital role in developing the unique quality of Liupao tea [46]. As described by Li [47], *Aspergillus* can change the volatile profiles of Liupao tea. Dominant microorganisms had different effects on the formation of tea quality. The results of potential toxigenic flora and citrinin toxin detected in Liupao tea supported this view [13]. It was necessary to detect the fungal community during the storage of Liupao tea to ensure the quality. 

### 4.3. Effectiveness of Multiplex PCR

In this study, the results of multiplex PCR species identification analysis showed that the effective judgment criteria for primer set 1 and primer set 2 were as follows. The purified strain was identified as *A. chevalieri* when two expected bands indicating *A. chevalieri* existed. Or it could be identified as *A. cristatus* when the expected bands of approximately 242 bp or 383 bp belonging to *A. cristatus* were observed. The *A. chevalieri* or *A. cristatus* contained in the microbial community can also be identified by multiplex PCR. Here, we recommend that the strains should be purified before multiplex PCR identification to improve the accuracy of identification. The accuracy of species identification could be improved by comparative analyzing the electrophoretic bands of two primers sets. [48] described that two pairs of primers designed based on orphan genes were effectively used to monitor the dynamic changes of *A. cristatus* during the processing of Fu brick tea. Multiplex PCR may be used not only for species identification, but also for quality monitoring of tea processing. The selected primers aOP4 and iOP2 can also identify *A. chevalieri* and *A. cristatus.* Previous studies have shown that dark tea contains a large number of tea polyphenols, the content of tea polyphenols in Liupao tea is 81.17 mg/g, and the content of tea polyphenols in Fu brick tea is 116.73 mg/g [49]. Endogenous polyphenols were found to inhibit PCR amplification [50]. The failure of multiplex PCR amplification in tea samples containing target microorganisms may be due to the high content of tea polyphenols. We recommend that microorganisms should be purified before identification.

Although the interference bands were not what we expected, they can partially indicate the genetic characteristics of the strain. The genetic diversity of strains can be inferred from the electrophoresis bands diversity of the multiplex PCR products. Electrophoresis bands have suggested that genetic diversity of *A. cristatus* isolated from Liupao tea and Fu brick tea. *A. cristatus* isolated from Liupao tea may be different from *A. cristatus* isolated from Fu brick tea. Morphological differences of *A. cristatus* isolated from Fu brick tea processed in different regions have been reported [51]. While the selected primers aOP4 and iOP2 results provide less information on genetic diversity.

### 4.4. Validity of Orphan Genes for Species Identification

Housekeeping genes are generally used for fungal identification. Currently, the ITS sequences are considered to be effective molecular sequences for identification at genus and species level [52]. However, the ITS genes of *A. cristatus* and *A. chevalieri* are highly conserved, which makes it impossible to distinguish the differences between strains in species level. Sequence specificity is a prerequisite for accurate species identification. Orphan genes are species-specific sequences [27]. In this study, we confirmed that orphan genes can be used for identification of closely related *Aspergillus* species. To our knowledge, this is the first report of application of orphan genes for the rapid identification of *Aspergillus* species. On the basis of this study, new species level identification methods based on multiplex PCR can be developed in the future.

The results of e-PCR experiments showed that the orphan gene primers for species identification also showed specificity in *A. niger* and *A. flavus*. These microorganisms have the potential to produce toxins. Some *A. niger* strains were found to produce the mycotoxins ochratoxin A and fumonisins [53,54,55,56]. *A. flavus* is a common contaminant in food, and about half of *A. flavus* strains produce aflatoxins [57]. This suggests that multiplex PCR based on orphan genes can be applied in food safety control. The multiplex PCR identification of toxigenic microorganisms can be carried out by combining conservative sequences of key toxigenic genes and species-specific orphan genes.

## 5. Conclusions

*A. cristatus*, *A. chevalieri*, and *A. pseudoglaucus* were identified as the dominant microorganisms in “golden flower” Liupao tea. To the best of our knowledge, this is the first report that dominant strains of “golden flower” fungi in Liupao tea were identified as *A. pseudoglaucus.* It is difficult to distinguish *A. cristatus* and *A. chevalieri* isolated from Liupao tea by ITS sequencing. Direct colony multiplex PCR was applied to closely related *Aspergillus* species identification in dark tea. Multiplex PCR specific primers sets containing four pairs of primers were developed to rapidly and reliably identify *A. cristatus* and *A. chevalieri* isolated from dark tea. Four pairs of species specific primers were designed based on orphan gene sequences of *A. cristatus* and *A. chevalieri*. Multiplex PCR results showed that orphan genes are specific, accurate and applicable to distinguish closely related *Aspergillus* species. The method has been proven to be efficient in species level identification of closely related *A. cristatus* and *A. chevalieri*. Based on the strategy of orphan gene sequence specificity, new species level identification methods can be developed in the future. This strategy provides a sequencing-free method for rapid species identification. This promising method will be greatly beneficial to the dark tea processing industry and consumers.

## Figures and Tables

**Table 1:** “Golden flower” Fungi identified and tea samples information.

ID	Fungi Isolated	Tea Sample	District	Manufacturer
L1	*A. chevalieri*	Liupao tea	Guangxi	Guangxi Wuzhou Tea Factory Co., Ltd.
L2	*A. chevalieri*	Liupao tea	Guangxi	Guangxi Wuzhou Maosheng Tea Co., Ltd.
L3	*A. cristatus*	Liupao tea	Guangxi	Guangxi Wuzhou Tea Factory Co., Ltd.
L4	*A. cristatus*	Liupao tea	Guangxi	Guangxi Wuzhou Maosheng Tea Co., Ltd.
L5	*A. pseudoglaucus*	Liupao tea	Guangxi	Wuzhou Chinatea Tea Co., Ltd.
L6	*A. pseudoglaucus*	Liupao tea	Guangxi	Wuzhou Chinatea Tea Co., Ltd.
H1	*A. cristatus*	Fu brick tea	Hunan	Hunan Yiqingyuan Tea Co., Ltd.
H2	*A. cristatus*	Fu brick tea	Hunan	Yiyang Guanlongyu Black Tea Development Co., Ltd.
H3	*A. cristatus*	Fu brick tea	Hunan	Chinatea Hunan Anhua First Tea Factory Co., Ltd.
H4	*A. cristatus*	Fu brick tea	Hunan	Hunan Baishaxi Tea Factory Co., Ltd.
H5	*A. cristatus*	Fu brick tea	Hunan	Yiyang Guanlongyu Black Tea Development Co., Ltd.
H6	*A. cristatus*	Fu brick tea	Hunan	Hunan Yiyang Tea Factory Co., Ltd.
H7	*A. chevalieri*	Fu brick tea	Hunan	Hunan Yiyang Tea Factory Co., Ltd.
H8	*A. cristatus*	Fu brick tea	Hunan	Hunan Yiyang Tea Factory Co., Ltd.
S1	*A. cristatus*	Fu brick tea	Shaanxi	Xianyang Jingwei Fucha Tea Co., Ltd.
Z1	*A. cristatus*	Fu brick tea	Zhejiang	Zhejiang Wuyi Camel Jiulong Brick Tea Co., Ltd.
M1	L2 + L3	Liupao tea	Guangxi	Artificial microbial communities
M2	L4 + L1	Liupao tea	Guangxi	Artificial microbial communities

**Table 2:** Primers designed in this study.

Name	Sequence	Expected Amplification Length/bp	Assigned Set
for *Aspergillus chevalieri*			
aOP1F	TTCGGCGGTATAGACTTCGTAAGACA	274	2
aOP1R	GGTGACCAAGTAGTAGGCAGCATCT		
aOP2F	CCTGTGAGGCTCTGGCGTAAGTATT	349	1
aOP2R	CTGCTCATCATCTTCCTGTCCACCA		
aOP3F	AGATCGCTCCACGATTCTGCTCTG	447	1
aOP3R	TTGGTTGCCAGTCTGCTGATAGGAA		
aOP4F	AACATGAACATCGACAGCCCACAAAG	659	2
aOP4R	GCATAGTCCTCCCGTCCAGTAAGC		
for *Aspergillus cristatus*			
iOP1F	CACCTGGAAGACCGACACCGAATC	242	1
iOP1R	TCATTGGCGAGTGGAAGGACAACAA		
iOP2F	ATGTCTCCAACCTTGTCCAGCACTT	383	2
iOP2R	TGATGTATCTGAGTTCGGCGAGAGTG		
iOP3F	ATCCGATGCCATTGTCTGTGTCTTG	529	1
iOP3R	GACCAGGCTATGGAACCTAACGAGAA		
iOP4F	GTCTAACTGCCACTGCTCGAATATGC	506	2
iOP4R	TCACTGACACTCTGCGAACGATACTT		

**Table 3:** Non-specific amplification of primers in e-PCR.

Primer ID	Genome Source	Non-Specific Amplification Length/bp
aOP2	*Aspergillus niger*	1,303,340
aOP2	*Aspergillus niger*	951,397
aOP2	*Aspergillus flavus*	2,083,709
aOP2	*Aspergillus fumigatus*	2,267,588
aOP4	*Aspergillus chevalieri*	1,739,704
iOP1	*Aspergillus flavus*	434,906
iOP1	*Aspergillus flavus*	205,828
iOP1	*Aspergillus fumigatus*	2,786,963
iOP1	*Aspergillus fumigatus*	3,164,056
iOP1	*Aspergillus nidulans*	37,955
iOP2	*Aspergillus flavus*	1,388,062
iOP2	*Aspergillus_cristatus*	1,716,279

## Data Availability

No new data were created or analyzed in this study. Data sharing is not applicable to this article.
